# ANGELITA HABR-GAMA. TRIBUTE TO A REMARKABLE WOMAN AND BRAZILIAN ICON OF GLOBAL COLORECTAL SURGERY. FORMER PRESIDENT OF THE BRAZILIAN COLLEGE OF DIGESTIVE SURGERY (2007–2008).

**DOI:** 10.1590/0102-67202025000031e1900

**Published:** 2025-09-15

**Authors:** Carlos Frederico Sparapan MARQUES, Fabio Guilherme CAMPOS, Carlos Walter SOBRADO

**Affiliations:** 1 Colon, Rectal and Anus Surgical Division, Coloproctology Discipline, Gastroenterology Department, Hospital das Clínicas, Faculty of Medicine, Universidade de São Paulo – São Paulo (SP), Brazil.

 The invitation to write an editorial about Professor Angelita Habr-Gama[Fig F1] was received with great joy, a deep sense of honor, and a strong sense of responsibility. For surgeons, the privilege of authoring this text is comparable to that of athletes attempting to describe the brilliance of Pelé, architects interpreting the distinctive lines of Niemeyer, or French scientists recounting the groundbreaking achievements of Madame Curie. 

**Figure F1:**
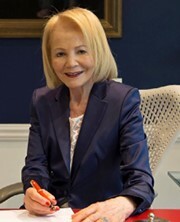


 A brief review of her 797-page *Curriculum Lattes* reveals an H-index of 66, more than 17,766 citations, and numer ous awards and honors conferred by nearly all surgical so cieties — national and international^6^. Such distinction has also attracted the attention of renowned biographers, including José Renato Nalini, José Pastore, and Ignácio de Loyola Brandão^7,8,11^. One might ask: what more could possibly be added? Drawing upon decades of professional, academic, institutional, and personal connections, as surgeons, former assistants, and long-standing colleagues, this editorial presents additional insights into her extraordinary influence. The aim was to briefly convey the breadth and impact of this remarkable surgeon and friend on the lives of countless doctors and non-doctors, both within Brazil and abroad. 

 Professor Angelita has been a true pioneer in many aspects of her life, consistently demonstrating exceptional proficiency in all her endeavors, as will be illustrated. The word *"no" has never been an option* in her life, and assertions that women were unfit for surgery only served to strengthen her resolve, ultimately leading her to become the most renowned and decorated Brazilian surgeon, both nationally and internationally^2^. 

## Origins and family

 The daughter of Lebanese immigrants, Angelita was born on Marajó Island, in the state of Pará. At the age of six, following the death of a brother due to appendicitis, her father, Kalil Nader Habr, sold the family’s livestock and farm in our suit of better living conditions and healthcare, and relocated the family to São Paulo. In their new city, Angelita attended the esteemed public schools Caetano de Campos and Presidente Roosevelt. Influenced by teammates on her volleyball team, she decided to pursue a career in medicine, despite initial resistance from her father, who had hoped she would become a teacher^4^ . 

## Academic career

 In 1952, Angelita was admitted in 8^th^ place to the medical program at the *Faculdade de Medicina da Universidade de São Paulo* (FMUSP), graduating in 1957. Her journey is marked by emotion from beginning to end. Although she initially had little interest in surgical practice, she possessed a natural talent for sewing, which she considered both a hobby and a skill. During an academic internship, her perspective shifted after she flawlessly sutured a patient’s abdominal wall. Defying the prevailing belief that Surgery was "unsuitable for women," she earned first place in the Surgery Residency Program at Hospital das Clínicas in 1958, becoming the first female resident in the Department. 

 Inspired by the 1960 International Congress of Coloproctology, held in São Paulo, she pursued further training at St. Mark’s Hospital in London, where she once again encountered resistance due to her gender. After two years of persistent ef fort, her determination prevailed, and she became the first fe male fellow at this esteemed institution. Upon returning to Brazil, she worked under the mentorship of Professor Alípio Corrêa Netto, who supervised her doctoral thesis and later served as best man at her wedding to Professor Joaquim Gama Rodrigues in 1964. 

 Angelita was a pioneer in the establishment of the "*Gastrão*" course and played a key role in the separation of the disciplines of Digestive Surgery and Coloproctology at Hospital das Clínicas, despite the challenges involved. She held several prominent positions, including Director of the Colon, Rectum, and Anus Surgery Division (1995–2001) and Head of the Department of Gastroenterology (1997–2001). In 1998, she was appointed Full Professor of the Colorectal Surgery Division at USP^4,6^. 

## Medical assistance and teaching

 Idealistic, rigorous, and disciplined, she consistently demanded the highest standards of herself and expected the same from those who worked alongside her in the care of her patients. These qualities were consistently evident in the operating room, during clinical rounds, academic meetings, thesis defenses, scientific publications, and at both national and international conferences. 

 Treating and respecting the patient has always been her guiding principle. When caring for a vulnerable patient, her perception of time often differed from that of her assistants. Personal dates — whether joyful or sorrowful — and the time of day were always secondary to the needs of those under her care. The intensity of the work never seemed to diminish her resolve. Even after long and complex procedures, she often emerged with renewed energy, surprising younger colleagues who were left exhausted. She made the difficult appear effortless, and, for her, it truly was. 

 Beyond medical and professional instruction, time spent with Professor Angelita has always served as an inexhaustible source of example and inspiration. Accordingly, this Editorial serves not only as a tribute, but also as an expression of sincere gratitude for the profound impact she has had on our lives and on the lives of countless others. 

## Medical societies

 Angelita dedicated a significant part of her career to medical societies, founding the Brazilian Group for Cancer Detection and Prevention (*Grupo Brasileiro para Detecção e Prevenção do Câncer* – BRADEPCA) in 1976. Her name appears alongside other prominent founding members of CBCD in the meeting minutes dated April 1, 1989. Her presidency during the 2007–2008 biennium was marked by strong advocacy for digestive tract surgery, consistently promoting its advancement and visibility^1,5,9^. She also served as National Vice President of the Brazilian College of Surgeons (*Colégio Brasileiro de Cirurgiões* – CBC) (1992–1994). Within the Brazilian Society of Coloproctology (*Sociedade Brasileira de Coloproctologia* – SBCP), she held multiple leadership roles, culminating in her election as President in 1981, an honor of particular distinction among coloproctologists. In 1995, she also assumed the presidency of the Latin American Society of Coloproctology (*Sociedade Latino-Americana de Coloproctologia* – ALACP)^4-6^. 

## Prevention and treatment of colorectal cancer

 Angelita made substantial contributions to the field of colorectal cancer and was among the pioneers in introducing colonoscopy in Brazil. She often recalled the excitement of removing her first polyp via colonoscopy, *personally interrupting* the adenoma–adenocarcinoma sequence. In 2004, she founded the Brazilian Association for the Prevention of Bowel Cancer (*Associação Brasileira de Prevenção do Câncer de Intestino* – ABRAPRECI) and coordinated the national Colorectal Cancer Prevention Program. Among her notable initiatives was the "Giant Colon," a traveling educational exhibit used globally to promote public awareness about the large intestine^4,6^. 

 Deeply concerned about patients undergoing rectal amputation whose anatomopathological specimens no longer showed evidence of neoplasia following neoadjuvant treatment, and inspired by the results of Norman Nigro in the management of anal cancer, Angelita revolutionized the treatment of rectal cancer. The introduction of the "Watch and Wait" approach allowed for the avoidance of surgery in patients who achieved a complete clinical response after chemotherapy and radiotherapy^3^. Although initially met with skepticism, this strategy is now a globally recognized practice in carefully selected cases^5,7,8,12^. The establishment of the International Forum on Rectal Cancer (*Fórum Internacional de Câncer Retal* – FICARE), held in São Paulo since 2007, became possible due to the international recognition of her leadership in the treatment of rectal cancer by coloproctologists worldwide. 

 Her dynamic personality, along with her innovative and groundbreaking contributions, has made Angelita a *celebrity* in colorectal surgery worldwide. She is frequently seen in selfies of speakers and attendees at the numerous courses, congresses, and medical events to which she is regularly invited. 

## Awards and honors

 Throughout her career, Angelita has received numerous honors, including being named an Honorary Member of several Coloproctology associations across South America, Europe, and the United States. In 2002, she became the first woman to join the prestigious American Surgical Association, established over a century ago. In 2017, she was the first surgeon to be featured on the cover of the renowned journal *Diseases of the Colon and Rectum*. In 2021, she was recognized as part of the top 2% of scientists whose work has had the greatest impact on the advancement of medical knowledge worldwide. 

 She was awarded the Bigelow Medal by the Boston Surgical Society — one of the highest distinctions in the field of surgery. This honor is not conferred annually, but rather only when the Executive Board of the Boston Surgical Society identifies a deserving recipient. In the Society’s 112-year history, only 33 individuals had received the award prior to her; Angelita became the 34^th^ recipient and the first female surgeon ever to be honored with this distinction^2,4,12^. 

 In 2024, she was inducted as an immortal member of the Academia Paulista de Letras^4^ and also became a member of the National Academy of Medicine. 

## Legacy for future generations

 Following in the footsteps of her mentors, Angelita continues to perform surgeries, conduct research, teach, and lecture. To this day, some students from FMUSP have been working with her since their second year of undergraduate studies. She affectionately refers to them as *gametas* (gametes). 

 Through what many consider divine intervention, and sustained by her remarkable physical and mental resilience, Angelita recovered from a severe COVID-19 infection in 2020. After a prolonged battle spanning several weeks in the ICU, she was discharged from the hospital, an event celebrated by her friends, family, assistants, students, and admirers. She was able to once again enjoy leisure time with her nephews, attend movies and theater performances, and savor the São Paulo cuisine she so deeply appreciates, always accompanied by her beloved Professor Joaquim Gama-Rodrigues. 

 She has often summarized her philosophy of life by stating that "*Success comes from hard work and sweat; the rest is talent, vocation, and intelligence.*" These qualities, and many others, have been present in abundance throughout every aspect of her remarkable life. 

## CONCLUSIONS

 Over the years, Angelita Habr-Gama has become one of the most distinguished living Brazilians, a singular figure in Medicine, Digestive Surgery, and Coloproctology, amassing extraordinary achievements throughout her life. It is fitting to conclude with the words of one of her favorite poets, Vinicius de Moraes, who wrote: "Life only yields itself to those who gave themselves"^10^

## Data Availability

The Informations regarding the investigation, methodology and data analysis of the article are archived under the responsibility of the authors.
